# Differential reductions in the capillary red-blood-cell flux between retina and brain under chronic global hypoperfusion

**DOI:** 10.1117/1.NPh.10.3.035001

**Published:** 2023-06-14

**Authors:** Baoqiang Li, Ji Leng, Ikbal Şencan-Eğilmez, Hajime Takase, Mohammed Ali H. Alfadhel, Buyin Fu, Mahnaz Shahidi, Eng H. Lo, Ken Arai, Sava Sakadžić

**Affiliations:** a Chinese Academy of Sciences, Shenzhen Institute of Advanced Technology, Brain Cognition and Brain Disease Institute; Shenzhen Fundamental Research Institutions, Shenzhen–Hong Kong Institute of Brain Science, Shenzhen, Guangdong, China; b Harvard Medical School, Massachusetts General Hospital, Athinoula A. Martinos Center for Biomedical Imaging, Charlestown, Massachusetts, United States; c Harvard Medical School, Massachusetts General Hospital, Department of Radiology, Charlestown, Massachusetts, United States; d Harvard Medical School, Massachusetts General Hospital, Department of Neurology, Charlestown, Massachusetts, United States; e University of Southern California, Department of Ophthalmology, Los Angeles, California, United States

**Keywords:** retinal imaging, brain imaging, capillary RBC flux, hypoperfusion, two-photon microscopy

## Abstract

**Significance:**

It has been hypothesized that abnormal microcirculation in the retina might predict the risk of ischemic damages in the brain. Direct comparison between the retinal and the cerebral microcirculation using similar animal preparation and under similar experimental conditions would help test this hypothesis.

**Aim:**

We investigated capillary red-blood-cell (RBC) flux changes under controlled conditions and bilateral-carotid-artery-stenosis (BCAS)-induced hypoperfusion, and then compared them with our previous measurements performed in the brain.

**Approach:**

We measured capillary RBC flux in mouse retina with two-photon microscopy using a fluorescence-labeled RBC-passage approach. Key physiological parameters were monitored during experiments to ensure stable physiology.

**Results:**

We found that under the controlled conditions, capillary RBC flux in the retina was much higher than in the brain (i.e., cerebral cortical gray matter and subcortical white matter), and that BCAS induced a much larger decrease in capillary RBC flux in the retina than in the brain.

**Conclusions:**

We demonstrated a two-photon microscopy-based technique to efficiently measure capillary RBC flux in the retina. Since cerebral subcortical white matter often exhibits early pathological developments due to global hypoperfusion, our results suggest that retinal microcirculation may be utilized as an early marker of brain diseases involving global hypoperfusion.

## 1 Introduction

The retina is regarded as an extension of the central nervous system. Many ischemic and neurodegenerative conditions that affect the brain may have manifestations in the retina.^1^ For example, brain ischemia, Alzheimer’s disease, Parkinson’s disease, and multiple sclerosis are associated with retinal microvascular abnormalities and retinal thickness reduction. These neurological disorders are also associated with ocular symptoms that are detectable through ophthalmological examinations;^2^^–^^7^ and the ocular changes often precede the conventional diagnosis of the symptoms in the brain.^6^^,^^8^^–^^17^ The diagnosis of these neurological disorders often involves expensive or invasive methods, such as MRI, PET, and cerebrospinal fluid assessment via spinal tap. Hence, retinal imaging may provide a low-cost and noninvasive alternative for the early diagnosis of neurological disorders.^1^

As the retinal and cerebral vasculatures share similar anatomical and physiological characteristics, it was hypothesized that the retinal microvascular imaging markers could be utilized to predict the emergence and development of cerebrovascular diseases.^18^ It has been reported that retinal microvascular abnormalities, e.g., decreased retinal arteriolar diameter and fractal size and increased venular diameter, were associated with a higher incidence of clinical stroke and stroke mortality, and this association was independent of the cerebrovascular risk factors, such as high blood pressure and diabetes.^19^^–^^22^ In addition to the acute vascular disorder (e.g., stroke), chronic cerebral hypoperfusion leads to white matter (WM) disease, which is a leading cause of ischemic stroke and vascular dementia in elders.^23^^,^^24^ The vulnerability of cerebral WM to global hypoperfusion is partially due to its distal location in the arterial blood supply.^25^^–^^27^ As a consequence of the prolonged insufficient blood supply, lesions are developed in the WM and can be diagnosed by the appearance of MRI hyperintensities.^28^ Moreover, WM disease is usually concomitant with cerebral small vessel disease—a progressive vascular disorder mainly affecting small vessels (e.g., arterioles, venules, and capillaries).^24^ However, no cure has been available to treat WM disease, and directly visualizing the brain microvascular changes with the existing neuroimaging techniques prior to the formation of WM lesions is technically challenging.^23^ Therefore, apart from the containment of the risk factors, a method for early diagnosis of WM disease is urgently needed.

As reported, retinal microvascular alterations were linked with cerebral small vessel disease and cognitive decline. For example, retinal microvascular morphological changes, such as smaller arteriolar diameter and fractal dimension, larger venular diameter, and increased arteriolar tortuosity, are associated with the presence of multiple cerebral microbleeds in human patients—a marker for cerebral small vessel disease.^29^ Similarly, in another study involving 3906 individuals, cerebral microbleeds and retinal microvascular abnormalities are associated with significant cognitive decline.^30^ Furthermore, it was found that reduced retinal microvascular branching complexity due to vascular remodeling was associated with cognitive impairment in human subjects.^31^ Taken together, these studies revealed a strong relation between retinal microvascular structural changes and cerebrovascular disorders. As shown in the previous studies, impaired cerebral blood flow (CBF) was strongly related to cognitive decline and development of various neurological disorders.^18^^,^^32^ Therefore, it is possible that the retinal microvascular blood flow changes can be utilized for early diagnosis of the brain disorders involving cerebral hypoperfusion, such as WM disease.

In this study, we applied two-photon fluorescence microscopy to investigate the retinal capillary red-blood-cell (RBC) flux changes due to chronic hypoperfusion in the mouse model of bilateral-carotid-artery-stenosis (BCAS). Our results showed that BCAS significantly decreased the retinal capillary RBC flux. Interestingly, the BCAS-induced reduction of capillary RBC flux in the retina was significantly larger than those in the cortical gray matter (GM) and subcortical WM as reported in our previous work.^27^ Since the subcortical WM often exhibits early pathological signs associated with chronic hypoperfusion, our results suggest that retinal microvascular blood flow may potentially serve as an early diagnostic marker for WM disease and other neurological disorders involving global cerebral hypoperfusion.

## 2 Material and Methods

### 2.1 Animal Preparation

**Fig. 1:**
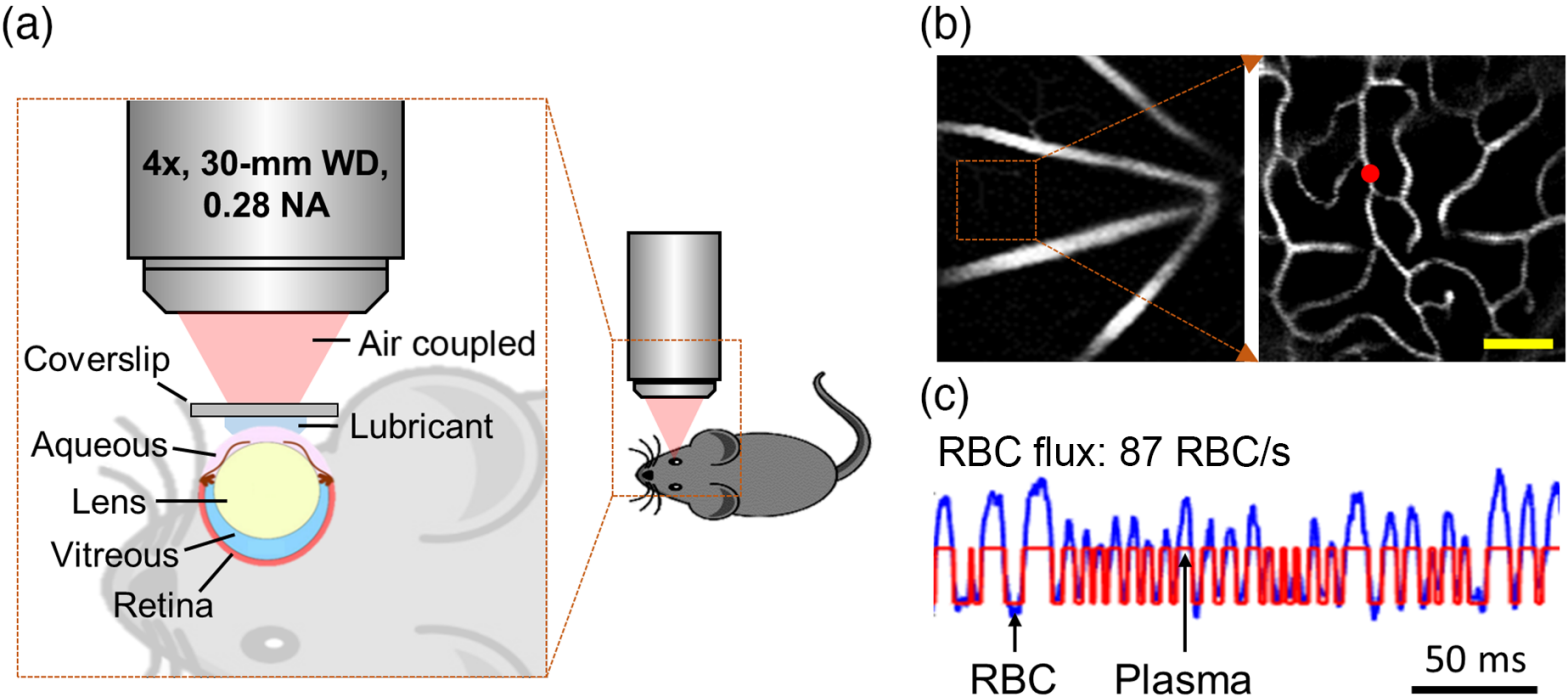
Measurements of capillary RBC flux in the retina. (a) Schematics of animal positioning. (b) Angiogram images acquired in the retina. The zoomed-in image on the right side was acquired approximately inside the region of interest enclosed by the square in the left image, but at a greater depth. Scale bar: 50  μm. (c) A representative fluorescence intensity time course (0.25-s-long trace) acquired in the capillary at the location denoted by the red dot in (b). The blue and red curves represent the experimental and fitted time courses, respectively. The “valley” and “peak” denoted by the arrows represent the RBC- and plasma-passages through the optical focal volume, respectively.

We used n=12 mice in this work (C57BL/6, female, 3 to 5 months old, 20 to 30 g; Charles River Laboratories). In this work, female mice were chosen to compare with our previous measurements in the brain, which were performed with female mice.^27^ The control and BCAS groups consisted of n=6 mice, while two BCAS mice were excluded due to failed surgery. BCAS was induced by placing the microcoils (0.18 mm in diameter) around both carotid arteries.^33^ The BCAS procedure was conducted 7 days prior to the imaging experiment. The details of the animal preparation for retinal imaging were described in our previous work.^34^ Briefly, a tracheotomy was conducted to control the animal respiration. Femoral artery cannulation was conducted to monitor the mean arterial blood pressure (MABP), collect the blood samples for blood-gas tests, and administer the contrast agent (e.g., Alexa680) into the bloodstream. Thirty minutes before imaging, tropicamide drops (1%) were applied to the right eye to dilate the pupil. The Hypromellose ophthalmic solution was subsequently applied over the cornea as eye lubricant, and then a microscope coverslip was placed over the cornea in contact with the lubricant gel. The animal position was adjusted such that optical axis of the imaging setup was perpendicular to the coverslip [Fig. 1(a)]. Throughout all the surgeries and experiments, the mice were anesthetized with isoflurane (1.5% to 2% during surgical procedures and 1% to 1.5% during experiments) and ventilated with a mixture of air and oxygen (${\mathrm{FiO}}_{2}$ of the gas mixture ∼25%); the body temperature was maintained at 37°C. For all the mice, the measurements were performed under normoxic normocapnia; physiological parameters, such as the MABP, systemic arterial partial pressure of oxygen ($P_{a}O_{2}$), systemic arterial partial pressure of carbon dioxide ($P_{a}{\mathrm{CO}}_{2}$), and pH, were recorded during experiments to ensure stable physiology. All the mice were euthanized after experiments.

All surgical and experimental procedures were approved by the Massachusetts General Hospital Institutional Animal Care and Use Committee (IACUC) in accordance with the National Institutes of Health’s Guide for the Care and Use of Laboratory Animals.

### 2.2 Imaging Setup

We employed a home-built two-photon microscope in this work.^35^^,^^36^ The imaging system was equipped with an InSight DeepSee ultrafast laser (tuning range: 680 to 1300 nm, ∼120  fs pulse width, and 80 MHz pulse repetition rate; Spectra-Physics). During imaging, the laser beam was raster-scanned with a pair of galvanometer mirrors (6215H; Cambridge Technology, Inc.) and was focused by an air-coupled objective lens (XLFLUOR4X/340, NA=0.28; Olympus). The axial translation of the objective lens was controlled by a motorized stage (M-112.1DG; Physik Instrumente) to image at different depths. The emitted fluorescence was selected by a band-pass emission filter (FF01-709/167-25; Semrock) and detected by a photon-counting photomultiplier tube (PMT) (H10770PA-50; Hamamatsu). The PMT current output was converted to voltage, discriminated by a voltage discriminator (C9744, Hamamatsu), and finally sampled by a digital acquisition board (NI PCle-6537; National Instruments).

### 2.3 Measurements of Capillary RBC Flux

To image the RBC passages, we labeled the blood plasma with a redshifted fluorophore – Alexa680 (2P-$\lambda_{\mathrm{exc}}=1280\;\mathrm{nm}$, $\lambda_{\mathrm{em}}=700\;\mathrm{nm}$) conjugated with 2-MDa dextran. Before imaging, the dextran-Alexa680 solution (0.1 ml at 5% W/V in PBS) was injected into bloodstream via the femoral artery cannula.

During imaging, we first positioned the imaging focal depth in the retina. Then, we manually selected the measurement locations inside most capillaries that could be visually identified in the survey image. The focused laser beam was parked at each selected location for 1 s. The detected fluorescence was digitally sampled and then binned with 250-μs-wide bins for postprocessing. The laser power and the duration of the laser “ON” phase during the 250-μs-long excitation was controlled by an electro-optic modulator. For retinal imaging, the laser power applied was <60  mW, which was within the power limit for ocular safety.^37^

RBC flux was estimated using our previously established method based on the RBC-passage modulated fluorescence intensity changes [Fig. 1(c)].^27^ The accuracy of the RBC flux calculation was evaluated by the coefficient of determination ($R^{2}$) between the experimentally measured and the numerically fitted fluorescence intensity time courses [Fig. 1(c)].^27^^,^^38^ In this work, data with $R^{2}\geq 0.5$ were kept for analysis.^27^^,^^39^

### 2.4 Statistical Analysis

The study design and reporting followed ARRIVE guidelines. All the data in this work are presented as mean ± STD, where applicable. Statistical comparisons were conducted using ANOVA. P≤0.05 was considered statistically significant. Sample sizes were sufficient to detect 30% difference between the mean values (coefficient of variance = 0.2, power = 0.8, α=0.05) and indicated in the text and figure legends, where relevant.

## 3 Results

### 3.1 Measurements of Capillary RBC Flux in the Mouse Retina

We first acquired the Alexa680-labeled angiograms [Fig. 1(b)] to guide selection of the measurement locations inside the capillaries. Figure 1(c) shows a representative 0.25-s-long fluorescence intensity time course that was acquired at the measurement location as denoted by the red dot in Fig. 1(b). The associated RBC flux was calculated as 87  RBC/s.

Physiological parameters were recorded during imaging to ensure the normal physiological condition of the animals. In the control group, averaging over n=6 mice, the MABP, $P_{a}O_{2}$, $P_{a}{\mathrm{CO}}_{2}$, and pH were 95±12  mmHg, 107±14  mmHg, 38±6  mmHg, and 7.39±0.04, respectively. In the BCAS group, averaging over n=4 mice, the mean MABP, $P_{a}O_{2}$, $P_{a}{\mathrm{CO}}_{2}$, and pH were 90±9  mmHg, 110±14  mmHg, 45±4  mmHg, and 7.39±0.03, respectively. The data were expressed as mean ± STD. No statistically significant differences in these parameters between the two groups were found.

### 3.2 BCAS Induces a Large and Significant Decrease in RBC Flux in the Retinal Capillaries

**Fig. 2:**
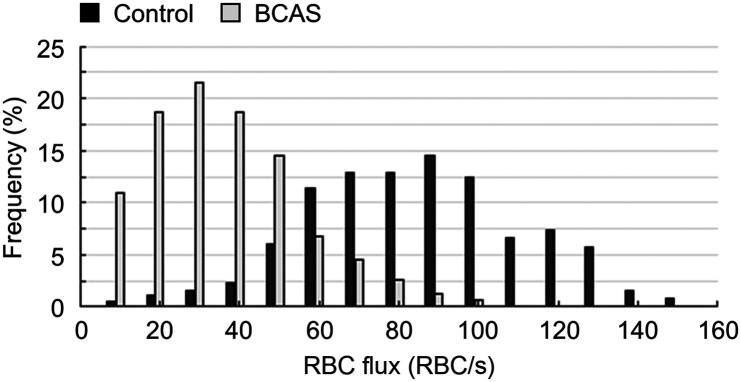
BCAS induces a significant decrease in the retinal capillary RBC flux. Distributions of RBC flux measured in the retinal capillaries in the control mice and in the mice subjected to BCAS. The measurements were performed in 381 capillaries across n=6 control mice and in 311 capillaries across n=4 mice subjected to BCAS.

The RBC flux acquired in the retinal capillaries under the controlled conditions spanned a large range of values, from ∼10 to ∼160  RBCs/s (Fig. 2). Importantly, under the BCAS-induced hypoperfusion, the retinal capillary RBC flux distribution was remarkedly shifted to a much lower range.

We also compared the capillary RBC flux changes in the retina with the similar measurements in the cerebral cortical GM and subcortical WM that have been reported in our previous work.^27^ The animal strain, sex, and age range associated with the measurements in the brain were the same as in this work; and the surgical and experimental procedures were almost the same except that a sealed cranial window was needed for brain imaging.^27^ In addition, no significant differences in the physiological parameters (e.g., MABP, $P_{a}O_{2}$, $P_{a}{\mathrm{CO}}_{2}$, and pH) were found between the different groups with the retinal and brain measurements.

We analyzed the incidences of low-flux capillaries with the data in Fig. 2 and in the previous work as well.^27^ As shown in Table 1, in the retina, the low-flux (e.g., ≤10  RBC/s) capillaries accounted for 0.6±1.4% of the total number of capillaries that were included into the analysis in the control mice; but this ratio increased to 2.8±2.4% in the mice subjected to BCAS, a more than four times increase in the incidence of low-flux capillaries. In comparison, the incidences of low-flux capillaries in the GM were 3.5±2.4% and 6.2±7.3% in the control and BCAS mice, respectively; and the incidences of low-flux capillaries in the WM were 4.8±9.4% and 4.0±4.4% in the control and BCAS mice, respectively. This analysis was also conducted with a cut-off flux value of ≤20  RBC/s. As shown in Table 1, induced by BCAS, the increase in the incidence of low-flux capillaries in the retina is still the most pronounced (e.g., a more than 13 time increase), in comparison to the counterparts in the GM and WM.

**Table 1:** Incidences of the low-flux capillaries (≤10 and ≤20  RBC/s) measured in the GM, WM, and retina, in the control and BCAS mice. The mean value in each group was first calculated over the capillaries of each mouse and then across mice in that group. Data are expressed as mean ± STD.

	≤10 RBC/s		≤20 RBC/s	
	Control	iBCASi	Control	iBCASi
Retina	0.6 ± 1.4%	2.8 ± 2.4%	1.1 ± 2.1%	14.4 ± 12.9%
GM	3.5 ± 2.4%	6.2 ± 7.3%	16.2 ± 16.7%	17.8 ± 20.5%
WM	4.8 ± 9.4%	4.0 ± 4.4%	8.8 ± 10.3%	15.1 ± 11.3%

**Fig. 3:**
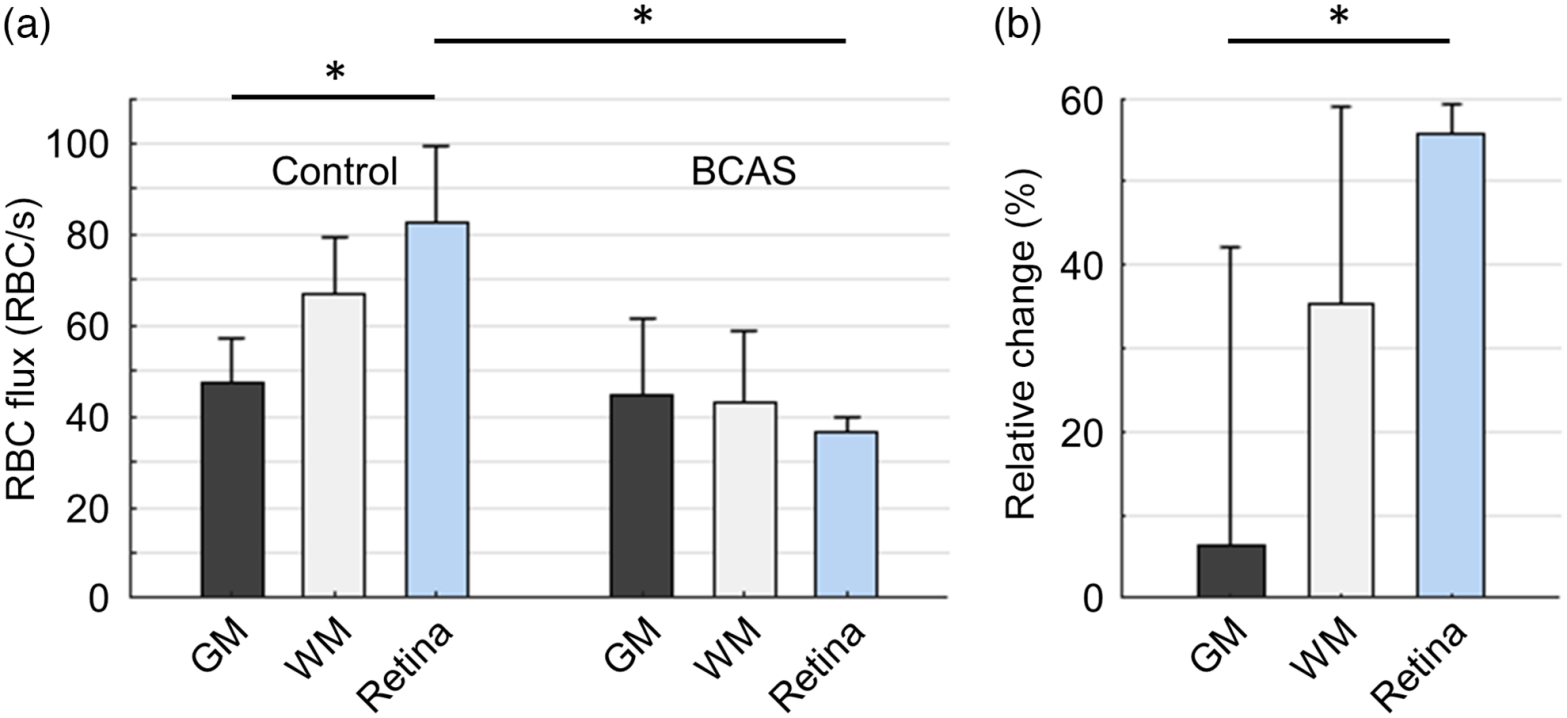
Comparisons of the BCAS-induced changes of capillary RBC flux in the cerebral GM, WM, and retina. (a) Mean capillary RBC flux in the cerebral cortical GM, subcortical WM, and retina. The measurements in the GM were performed in 343 and 262 capillaries, across n=5 control mice and n=4 mice subjected to BCAS, respectively; the measurements in the WM were performed in 191 and 133 capillaries, across n=5 control mice and n=4 mice subjected to BCAS, respectively.^27^ The measurements in the retina are the same as in Fig. 2. The mean value in each group was first calculated over the capillaries of each mouse and then across mice. The comparisons were carried out using two-way ANOVA followed by the Tukey HSD *post* *hoc* test. The asterisk symbol (*) indicates statistical significance (P<0.05). (b) The BCAS-induced relative changes of capillary RBC flux in the GM, WM, and retina. In each group (e.g., GM, WM, or retina), the relative RBC flux change corresponding to each BCAS animal was calculated as the ratio of the difference between the mean control RBC flux value averaged over all the control animals in that group and the RBC flux value of that specific BCAS animal to the mean control RBC flux value averaged over all the control animals in that group. The comparisons were carried out using one-way ANOVA followed by the Tukey HSD *post hoc* test. The asterisk symbol (*) indicates statistical significance (P<0.05). Here, we applied a linear mixed-effects model to evaluate the effects of the measurement locations (GM vs. WM vs. retina) and conditions (control vs. BCAS) on the comparisons between the different groups with capillary number per animal as a random variable. We found that the measurement locations (GM, WM, and retina) and conditions (control and BCAS) had significant impacts on the comparisons. Data are expressed as mean ± STD.

Next, we compared the mean capillary RBC flux in the brain and retina. We previously found that under the controlled conditions the mean capillary RBC flux in the cerebral subcortical WM (67±12  RBC/s) was significantly higher than that in the cerebral cortical GM (48±10  RBC/s).^27^ Here, we further found that under the controlled conditions the mean capillary RBC flux in the retina (83±17  RBC/s) was higher than in the GM and WM, and the comparison of the mean capillary RBC flux between the retina and GM was statistically significant [Fig. 3(a)].

Besides, as shown in our previous work, the BCAS-induced decrease in RBC flux in the WM capillaries was larger than that in the GM capillaries.^27^ Importantly, we further found that the BCAS-induced decrease in RBC flux in the retinal capillaries was much larger than that in the WM capillaries. Specifically, as shown in Fig. 3(a), the mean RBC flux in the retinal capillaries decreased significantly from 83±17  RBC/s under the controlled conditions to 37±3  RBC/s under the BCAS-induced hypoperfusion; whereas the mean RBC flux in the WM capillaries decreased from 67±12  RBC/s under the controlled conditions to 43±16  RBC/s under the BCAS-induced hypoperfusion, a much smaller decrease than what was observed in the retinal capillaries; while the decrease in RBC flux in the GM capillaries was subtle (from 48±10 to 45±17  RBC/s).

Last, we analyzed the BCAS-induced relative change of RBC flux in each group. Specifically, the relative decrease in RBC flux in the retina, WM, and GM, were 56±43%, 36±24%, and 6±36%, respectively [Fig. 3(b)]. This result indicates a strong trend of larger decrease in capillary RBC flux in the retina than in the GM and WM. The statistical test between the three groups shows that the relative decrease in capillary RBC flux in the retina is significantly larger than in the GM.

## 4 Discussion

We have obtained a large number of RBC flux measurements in the retinal capillaries (in total 692 capillaries across n=10 mice). Due to the redshifted two-photon excitation (e.g., 1,280 nm) and emission (e.g., 700 nm) spectrums of Alexa680, large-etendue detection optics, and improved fluorescence detection sensitivity with a photon-counting PMT module, imaging of capillary RBC flux down to the cerebral subcortical WM in live mouse has been enabled.^27^ In this work, we applied the same technique to measure efficiently capillary RBC flux in mouse retina. The accuracy of RBC flux calculation was evaluated by the $R^{2}$ value calculated between the measured and the numerically fitted fluorescence intensity time courses. As in our previous work,^27^^,^^39^ the data with $R^{2}\geq 0.5$ were kept for analysis. In this work, each capillary was scanned for 1 s, which was consistent with our previous work.^27^ The 1-s-long scans are expected to smooth out the cardiac and, to some extent, respiratory effects on the capillary blood flow fluctuations. In addition, the RBC flux measurements might be affected by an aliasing effect that was typically induced by high flow speed. By comparing the data presented in this work with the previously reported measurements, capillary RBC flux was much higher in the retina^40^^,^^41^ than in the brain,^27^^,^^38^ thus, the aliasing effect might impose a stronger impact on the retinal RBC flux measurements. Here, our previous work indicated that a temporal resolution of Δt=1.5  ms was sufficient to measure RBC flux up to 150  RBC/s without any aliasing effect.^42^ With Δt=250  μs used in this work, we expect a much higher upper limit of the RBC flux detection. Hence, the aliasing effect is likely not a factor for the retinal RBC flux measurements presented in this work.

Other imaging techniques for measuring the retinal capillary blood flow parameters have also been demonstrated. For example, optical coherence tomography (OCT) angiography-based method features a fast and label-free detection of microvascular blood flow, but it only provides a qualitative estimation instead of the absolute measurement of capillary flow parameters, hindering its application when comparing across subjects.^40^^,^^43^ By taking advantage of the RBC-passage induced OCT signal changes, retinal capillary RBC flux can be quantitatively measured.^44^ However, the intrinsic light contrast is typically associated with a suboptimal signal-to-noise ratio (SNR), affecting the accuracy of RBC flux calculation.^40^^,^^43^^–^^45^ Other label-free techniques for measuring retinal capillary blood flow, such as phase-contrast scanning light ophthalmoscopy and near-confocal microscopy, have also been developed.^40^^,^^43^^,^^45^^–^^47^ The phase-contrast technique is capable of capturing the two-dimensional spatial image of individual RBCs within a single capillary,^40^^,^^43^ so the RBC flux can be directly measured. The line-scan technique that is typically combined with fluorescence labeling^48^ can also be implemented in a label-free fashion with scanning light ophthalmoscope or near-confocal microscopy.^45^^–^^47^ With this technique, the angle and the number of the RBC-passage-induced bright stripes in the spatiotemporal image are extracted to calculate RBC velocity and flux, respectively. However, the phase-contrast or line-scan imaging is usually conducted in a small lateral field of view (e.g., covering only the targeted capillary), which limits the data acquisition to one capillary segment at a time, making large sampling impractical.^40^^,^^43^^,^^45^^–^^47^ Indeed, as shown in those previous works, less than 10 capillaries per animal were measured. RBC flux can also be measured by imaging the fluorescently labeled RBCs (fRBC) with confocal microscopy.^49^^–^^51^ With this technique, a portion of blood is withdrawn from the donor animal, the fRBCs are subsequently prepared and then injected back into the bloodstream of the animal. While this procedure potentially enables higher image contrast and better SNR compared to the label-free counterparts, fluorescence labeling of a fraction of the body RBCs inevitably causes underestimation of the RBC flux. In comparison, our technique demonstrated its advantages in the aspects of high SNR and sampling efficiency, and it may be easily adopted with a standard commercial two-photon microscope.

The retina, as the embryological projection of forebrain,^52^ shares a similarly high metabolic demand.^53^ In this work, we found that in the control mice the mean retinal capillary RBC flux was 83±17  RBC/s. Previous works reported the mean capillary RBC flux ranging from 60 to 100  RBC/s, using scanning light ophthalmoscope^40^^,^^43^ and confocal microscopy with fRBC.^50^^,^^51^ The mean retinal capillary RBC flux under the controlled conditions presented in this work is consistent with this range.

We further found that in the control mice, capillary RBC flux in the retina (83±17  RBC/s) was statistically higher than in the cerebral GM (48±10  RBC/s) that has been reported in our previous work.^27^ Direct comparison between the retinal and cerebral microcirculation (e.g., using the same animal model, similar animal preparation and under similar, well-controlled experimental conditions) was previously lacking. Comparing across different studies, capillary blood flow in the retina seemed to be much higher than in the brain and the other peripheral microvascular networks, e.g., skin, cremaster muscle, and mesentery.^40^^,^^44^^,^^49^^–^^51^ The reason for such a high retinal capillary blood flow may be due to a very high retinal energy demand as compared with other organs, which requires maintaining the function of mammalian retina at its upper limit of oxygen supply.^54^^,^^55^

It has been reported that the mouse model of BCAS induced an initial decrease in CBF to 50% to 60% of the baseline value, followed by a gradual recovery of CBF over 1 week to ∼70% of the baseline, which might last for weeks or months.^33^ This model is associated with gradual WM damage and behavioral deficits. However, the myelin damage, loss of oligodendrocytes, and cognitive deficits are typically not significant during the first week after the BCAS induction.^33^^,^^56^^–^^59^ Therefore, 7 days post BCAS may still represent an early phase of the BCAS-induced pathological process in the brain. As a variant of BCAS, the bilateral carotid artery occlusion (BCAO) model induces an acute and much severer shortage of CBF and has been utilized for the studies of ocular ischemic syndrome. Several studies with rodent models have demonstrated that ischemia associated with BCAO would result in retinal hypoxia, leading to vascular remodeling, reduced b-waves in electroretinography, photoreceptor and retinal ganglion cell degeneration, thinning of the retinal tissue, and eventually loss of pupillary reflex, impairing the visually guided behavior.^60^^–^^72^ On the other hand, it has been reported that BCAS model, unlike the BCAO model, did not exhibit optic nerve damage and were therefore suitable for behavioral experiments.^73^^,^^74^ Although no data on the BCAS-induced retinal tissue oxygenation are available, several studies have reported reduced retinal vascular density in subjects with carotid stenosis.^75^^–^^78^ Indeed, a recent study established a relationship between retinal vascular perfusion and cerebral hemodynamics in subjects with internal carotid artery stenosis.^79^

Our results indicate that the retinal capillary RBC flux acquired 7 days post the BCAS induction was reduced by 55.7%, a remarkably greater relative reduction comparing with those observed in the cerebral WM (35.5%) and GM (6.4%), of which the measurements were also performed 7 days post BCAS.^27^ Previous studies reported that the cerebral WM exhibited a differential vulnerability to global hypoperfusion as compared with the cerebral GM.^27^^,^^80^^,^^81^ The results in this work further demonstrate that microvascular blood flow in the retina may be more sensitive to the global hypoperfusion as compared with the brain. A potential explanation for this observation is that BCAS may have caused the blood redistribution from the carotid arteries in a way that favored the blood supply to the brain needed for survival. In fact, clinical evidence indicated that flow direction in the ophthalmic arteries was reversable under acute and chronic hypoperfusion, acting as a mechanism for securing CBF, but at the expense of increased risk for retinal ischemia.^82^^–^^88^ Another possibility is the differences between the retina and the brain in collateral vascular networks that compensate for the deficiency of the primary supply conduit.

Several limitations exist in this work. First, isoflurane-anesthesia-induced variation of systemic physiology between animals would be confounding factor to the measurements. Since key physiological parameters (e.g., MABP, $P_{a}O_{2}$, and $P_{a}{\mathrm{CO}}_{2}$) during imaging were not significantly different between animal groups, we expect that the impact of anesthesia on the measurements in different animal groups (e.g., BCAS versus controls) was minimized. In addition, the surgical preparation-induced inflammatory response might potentially affect the systemic hemodynamics, and this impact might be differential in the brain versus in the retina. Moreover, some differences in the surgical procedures between groups (e.g., no craniotomy for retinal imaging, and no sham operation for the control group to compare with the BCAS group) potentially affected the group comparisons too. However, the potentially differential effects of anesthesia, inflammatory response, and surgical procedures on the measurements have not been well studied previously and need to be better addressed in the future. In this work, female mice were chosen for comparison with our previous measurements performed in the cerebral cortical GM and subcortical WM of the female mice.^27^ While this experimental design enabled more appropriate group comparison, future studies should address the effect in the male mice. Furthermore, we used the 2-MDa dextran-conjugated Alexa680 probe for the retinal measurements. While it has been shown that this large molecule might affect blood flow in the anesthetized mice,^89^ this selection was made intentionally to better compare the results in this work with our previous measurements in the brain, which were also performed with the same 2-MDa probe.^27^ Besides, it has been reported that capillary stalling would be increased under the acute animal preparation with isoflurane anesthesia in control animals,^90^ as well as in the animal models of abnormal brain conditions, such as stroke, vascular disease, and Alzheimer’s disease.^91^^–^^93^ The capillary stalling phenomena might influence the detection of RBC passages and affect the evaluation of the RBC flux measurements. However, the differences between the observed BCAS-induced relative decrease of capillary RBC flux in the retina, cerebral cortical GM, and subcortical WM were much larger than the reported capillary stalling incidence in the brain after acute surgical procedures (e.g., <10%^90^^,^^92^). Therefore, we expect that capillary stalling did not affect the conclusions made with our measurements. Nevertheless, it would be important to consider this effect in the future studies. Last, adaptive optics (AO) techniques have been introduced in retinal blood flow imaging to correct the aberration brought by the optics of the eye. However, AO is typically associated with a small imaging field of view and may add substantial complexity to the imaging system and imaging practice. While two-photon imaging of RBC flux in a sparse capillary network in mouse retina does not require AO, applying AO techniques to correct the aberrations in the mouse eye may enhance assessment of capillary RBC flux in individual overlapping retinal layers and improve the resolution of the retinal angiograms, which are critical for morphological analysis.^45^^–^^47^^,^^94^

In conclusion, we demonstrated a method for *in vivo* measurements of capillary RBC flux in mouse retina using two-photon microscopic imaging of fluorescence-labeled RBC passages. This method is easily adopted with a standard two-photon microscope and features a high sampling efficiency. We found that retina exhibited a higher capillary RBC flux under normal physiological conditions but was much more vulnerable to global hypoperfusion, as compared with the cerebral cortical GM and subcortical WM. This finding suggests that assessment of the retinal microcirculation has potential as an early diagnostic marker of ischemic conditions in the brain. Further studies are necessary to better understand the link between retinal and cerebral hypoperfusion. Moreover, application of clinical and research imaging methods for assessment of retinal capillary perfusion, such as OCT angiography and AO retinal imaging, may potentially provide information about cerebral hypoperfusion in humans.
